# Identification of the Perturbed Metabolic Pathways Associating With Renal Fibrosis and Evaluating Metabolome Changes of Pretreatment With Astragalus polysaccharide Through Liquid Chromatography Quadrupole Time-Of-Flight Mass Spectrometry

**DOI:** 10.3389/fphar.2019.01623

**Published:** 2020-01-29

**Authors:** Lei Ren, Xiao-Ying Guo, Fei Gao, Mei-Li Jin, Xiang-Nan Song

**Affiliations:** ^1^ Department of Clinical Laboratory, Affiliated Hospital, Guilin Medical University, Guilin, China; ^2^ Department of Clinical Laboratory, Daqing Oilfield General Hospital, Daqing, China; ^3^ Department of Clinical Laboratory, The Second Affiliated Hospital of Harbin Medical University, Harbin, China

**Keywords:** metabolomics, biomarker, metabolic pathway, liquid chromatography quadrupole time-of-flight mass spectrometry, metabolome

## Abstract

Renal fibrosis is glomerulosclerosis and renal tubulointerstitial fibrosis caused by the increase of interstitial cells and intercellular substances and the accumulation of extracellular matrix, and is a common pathological manifestation of renal disease progressing to end-stage renal failure. It has proved that Astragalus polysaccharide (AP) has curative effect on renal disease; however, its therapeutic mechanism on renal fibrosis is still unclear. Metabolomics approach provides an opportunity to identify novel molecular biomarkers. The purpose of this study is to study the changes of serum metabolic profile of rats with unilateral tubal ligation and replication of renal fibrosis model and the therapeutic effect of AP on it. The blood samples of rats in the control group, renal fibrosis model group, and AP treatment group collected on the 21st day were analyzed by metabolomics method based on UPLC-Q-TOF-MS. Principal component analysis (PCA) showed that clustering was obvious and significantly separated, and paired partial least squares discriminant analysis (OPLS-DA) was used for further analysis. Combined with the network databases such as HMDB and KEGG and a large number of literatures, 32 potential biomarkers related to renal fibrosis were preliminarily screened out and further verified by MS/MS secondary debris information. After pretreatment with AP, 20 biomarkers were significantly regulated, and correlated with phenylalanine, tyrosine, and tryptophan biosynthesis, phenylalanine metabolism, arachidonic acid metabolism, etc. It also revealed the metabolic changes of renal fibrosis and intervention effect of AP. These data uncover a link between metabolism and the molecular mechanism with potential implications in the understanding of the intervention effect of AP. Conclusively, UPLC-Q-TOF-MS–based metabolomics can be valuable and promising strategy to understand the disease mechanism and natural drug pretreatment.

## Introduction 

Renal fibrosis refers to glomerulosclerosis and tubulointerstitial fibrosis caused by the increase of interstitial cells and interstitial cells under the action of various pathogenic factors such as inflammation, injury, drugs, etc., especially the increase of matrix protein synthesis and the inhibition of matrix degradation resulting in a large accumulation of extracellular matrix (Zeisberg et al., 2002). It is a common pathological manifestation of renal disease progression to end-stage renal failure (Qin et al., 2011). In recent years, with the deepening of research, it has been found that renal tubulointerstitial fibrosis is related to the conversion or transdifferentiation of various cells (such as interstitial fibroblasts, renal tubular epithelial cells, etc.) into myofibroblasts (MFBs), and the transdifferentiation mechanism is also an important mechanism of fibrosis (Liu, 2006; Min, 2010). Fibroblasts are one of the main intrinsic renal cells in the stroma and the most important extracellular matrix secreting cells in the process of renal interstitial fibrosis, thus playing an important role in the process of disease. At present, it is believed that the molecular mechanism of renal fibrosis is mainly divided into four stages: 1) Activation and damage of cells. Inflammatory injury causes activation of renal tubular epithelial cells and infiltration of monocytes and macrophages in renal interstitium. 2) Release of fibrogenic factors. It includes cytokines, growth factors, vasoactive factors, chemotactic adhesion factors, etc (Zeisberg and Kalluri, 2004). 3) Formation of fibrosis. The main manifestation is that matrix protein synthesis increases and degradation decreases, resulting in matrix protein deposition in renal interstitium. The degradation of matrix protein is mainly affected by some protease inhibitors, which can inactivate renal protease (Vasko, 2016). 4) Renal structure and function are damaged. The structural and functional damage of kidney is mainly caused by ECM deposition in kidney (Eddy, 1996; Liu and Youhua, 2011). However, most of the existing researches on renal fibrosis focus on molecular mechanisms, and there are few reports on changes in metabolic levels. Changes in endogenous metabolites may provide basis for revealing the pathogenesis and early diagnosis.

Metabolomics has the comprehensive qualitative and quantitative analysis of metabolites in the body, the dynamic changes in different environments such as normal living conditions and internal and external environmental changes are described (Weckwerth, 2003). Living organisms are affected by diseases, toxicity, genes, or environmental factors, and the content of endogenous small molecules in the body will be correspondingly upregulated or downregulated. The purpose of metabolomics is to find the affected small molecules and finally apply them to the diagnosis and drug screening of some related diseases (Fiehn, 2002). As an application-driven emerging science, it has been widely used in medicine-related fields (Wang et al., 2013; Qiu et al., 2017). Non-targeted metabolomics refers to non-biased detection of the dynamic changes of all small molecule metabolites before and after stimulation or disturbance in cells, tissues, organs, or organisms, and screening of differential metabolites through bioinformatics analysis, and pathway analysis of differential metabolites to reveal the physiological mechanism of their changes (Dettmer et al., 2010; Zhang et al., 2014; Zhang et al., 2017). Targeted metabolomics focuses on a specific metabolic pathway or a specific metabolic pathway, and analyzes these specific components to confirm whether these components are different substances (Zhang et al., 2018). Metabolomics is not only applied to diseases and general health, but also has great application space in agronomy, environment, and nutrition. In recent years, with its high throughput, high resolution, high accuracy, and other characteristics, it has provided new ideas and methodological guarantees for the development and improvement of modern research of traditional Chinese medicine (TCM) and the diagnosis of chronic diseases (Zhang et al., 2010; Sun et al., 2013; Song et al., 2017).

Astragalus membranaceus (AM) has a long history of medicinal use and is an important qi-invigorating drug. Li Shizhen describes it as the “longest tonic” in the *Compendium of Materia Medica*. It is one of the most commonly used TCMs in past dynasties (Shao, 2004). There are many reports that AM or TCMs with AM as the main drug can improve renal function of patients with chronic renal failure (Chang et al., 2012). Recent studies have shown that the main chemical components of AM include Astragalus polysaccharides (AP) (Liu et al., 2010), saponins, flavonoids, and amino acids (Shao, 2004). Modern pharmacological research shows that AM can enhance and regulate the body’s immune function (Adesso et al., 2018), and has the effects of dilating blood vessels, improving microcirculation, reducing plasma osmotic pressure and blood viscosity, regulating lipid metabolism, etc (Cho and Leung., 2007). In addition, it can also increase renal blood flow, which is conducive to the elimination of harmful substances such as lipid peroxide, and reduce the deposition of lipid in glomeruli and renal interstitium and the formation of microthrombi, thus protecting the kidney (Song et al., 2009; Li et al., 2011). Renal fibrosis is the terminal end of the development of various renal diseases, which will lead to irreversible damage to renal function. However, there is still a lack of therapeutic drugs that can inhibit or even reverse renal fibrosis. In recent years, TCM has shown its unique advantages with multi-channel and multi-target treatment strategies. AP is the active ingredient with the highest content in AM, which has the effects of lowering blood sugar, reducing proteinuria, regulating lipid metabolism, and improving renal function in patients with nephropathy (Lian et al., 2014). Therefore, this study uses high-throughput metabolomics to study the effect of AP on endogenous metabolites in renal fibrosis rats and to find potential biomarkers, providing a method for clinical diagnosis of metabolic level of renal fibrosis, and further providing a basis for studying the mechanism of improvement of AP on renal fibrosis.

## Experiments

### Drugs and Reagents

AP (80%): Nanjing Zelang Pharmaceutical Technology Co., Ltd. (Nanjing, China) batch number: ZL120513. Acetonitrile was purchased from Merck Corporation (Merck, Germany). Methanol was purchased from Fisher Scientific Corporation (Loughborough, UK). Leucineenkephalin was obtained from Sigma-Aldrich (St. Louis, MO, USA).

### Animal Grouping and Model Replication

Animal experiments were conducted in accordance with the guidelines for animal experiments of Affiliated Hospital of Guilin Medical University and approved by the Animal Ethics Committee. Thirty SPF healthy female SD rats (weighing 200–220 g) were purchase by the Experimental Animal Center of Affiliated Hospital of Guilin Medical University. Rats were raised in a standard environment (temperature: 18–25°C, humidity: 45–60%, light/dark cycle alternating every 12 h). During the whole experiment, rats were allowed to eat freely and take water. Before the start of the formal experiment, all animals adapted to the environment for 1 week, and then the model was reproduced by unilateral ureteral ligation. All rats were randomly divided into sham operation group (control group) with 10 rats and operation group (model group and treatment group with 10 rats each). After anesthesia, rats were shaved locally by intraperitoneal injection of 5% chloral hydrate. After routine disinfection, abdominal midline incision was selected. Open skin to abdominal cavity, free kidney and ureter in turn. Left ureter was held up at the middle part with tissue forceps, hemostatic forceps was clamped. Left ureter was ligated twice at both ends with 4-0 silk thread near renal pelvis. Ureter was cut off, and skin was continuously sutured. The surgical approach of the control group was the same as that of the operation group. After entering the abdominal cavity, the left ureter was separated but the ureter was not ligated, and then the skin was sutured. The administration group was given AP aqueous solution (1,000 mg·kg^−1^·d^−1^, 2 ml/d) by gavage, while the other groups were given the same amount of normal saline for 21 days.

### Collection and Preparation of Samples

Twenty-four hours after the last administration, weigh and record the weight of each group of rats, inject 0.5 ml/100 g of 4% chloral hydrate solution intraperitoneally to anesthetize the rats, blood was taken from abdominal aorta quickly, left to stand for 30 min, centrifuged at 4,000 rmp for 15 min at 4°C, supernatant was taken and split into five parts, 1 ml for each part, for detection of serum creatinine (SCr), blood urea nitrogen (BUN), and blood metabolomics, and freeze-store at −80°C till analysis. Double kidneys were weighed, and the kidney–body ratio (2 × left kidney/body weight) was calculated.

The unfreeze the serum sample in an ice bath, take 200 μl of serum sample, add 800 μl of acetonitrile to precipitate protein, vortex for 30 s, 4°C, 13,000 rmp for 15 min, remove supernatant, blow-dry in a 40°C water bath under nitrogen, add 200 μl of methanol:water (9:1, v/v) mixed solution to the residue, vortex for 60 s, centrifuge at 4°C, 13,000 rmp for 10 min, take supernatant through a 0.22 μm filter membrane, and take 5 μl of filtrate for UPLC-MS/MS analysis.

### Mass Spectrum Conditions

The UPLC-MS/MS analysis was carried out using a Waters ACQUITY™ ultra performance liquid chromatography system (Waters Corp., Milford, USA) coupled with a Waters Synapt™ Q-TOF Mass system (Waters Corporation) equipped with an ESI ion source and hybrid Q-TOF mass spectrometer in both ESI− and ESI+ ion mode. The UPLC column was a Waters BEH C18 (2.1 × 100 mm, 1.7 μm), and flow rate is 0.4 ml/min with 40°C temperature. The mobile phases A and B are respectively acetonitrile with 0.1% formic acid and water with 0.1% formic acid and optimal linear elution gradient procedure as followed: 0–1 min, 2–20% A; 1–3 min, 20–40% A; 3–10 min, 40–80% A; 10.0–13.0 min, 80–90% A; 13.0–14.0 min, 90–98% A; 14–15 min, 98–2% A; 15–17 min, 2% A. All samples were kept in a refrigerator at 4°C during analysis.

Mass spectrometry conditions: ESI− and ESI+ ion scanning mode capillary voltage respectively 1,500 and 1,300 V. All other parameters are the identical, respectively sample cone voltage: 60 V; ion source temperature: 110°C; desolvation gas temperature: 350°C; desolvation gas flow: 800 L/h; the flow rate: 20 L/h. The calibration solution is leucine enkephalin ([M+H]+ = 556.2771; [M+H] − = 554.2615), injected at an injection amount of 100 μl/min every 15 s, and the collection range is 100–2,000 Da. All samples were collected and collected under the control of Masslynx 4.1 software.

### Multivariate Data Processing

The established analysis method is used to carry out full scanning of ESI+ and ESI− of the sample to obtain the metabolic profile map containing three-dimensional information of individual samples of the sample group. Peak identification and matching are carried out by MassLynx V4.1 software. After data extraction and standardization, principal component analysis (PCA) of unsupervised mode is firstly carried out by EZinfo 3.0.3 software, and PCA score chart and 3D-PCA score chart reflecting aggregation and dispersion degree between groups are drawn. In order to find endogenous metabolites that play a key role in metabolic profile, paired partial least squares discriminant analysis (OPLS-DA) was performed on metabolic profile data between control group and model group to obtain VIP plot and loading plot. The further away from the center the ion pair contributes to its grouping. According to the variable weight value (VIP) of the relevant reactive ion contribution degree, in the VIP scatter plot, the ion fragments are arranged in a V-shape, the bottom ions (VIP value is small), the contribution to differential metabolism is smaller; top ions (VIP value is large) contribute a lot to the changes of metabolic profile trajectory. In loading plot, the farther away from the distant point in the load map, the greater the contribution of ions to the change of metabolic profile trajectory. In order to verify whether the differences found in multi-dimensional statistics are statistically significant, T-Test was used to screen metabolites with greater difference between groups. Finally, VIP > 1 and p < 0.05 were selected as screening conditions as potential biomarker sets. According to the accurate retention time and mass–core ratio of the obtained potential biomarkers, the result of matching metabolite database under the metabolite identification module of Progenesis QI software is fitted. The higher the score is, the greater the accuracy of structural identification. The obtained results are marked, and then retrieved by HMDB (http://www.hmdb.ca/), KEGG, (http://www.genome.jp/kegg/), and other databases for secondary matching. Finally, the structure analysis and verification are carried out by MS/MS data to determine the name and chemical structure of the biomarker. The mass fragment module in Masslynx V4.1 software was used to determine the possible cleavage information of the marker. Then, the identified biomarker information is input to the MetaboAnalyst for relevant metabolic pathway matching to obtain the blood metabolic pathway related to the renal fibrosis model.

### Statistical Methods

Statistical analysis was performed on each group of data using the SPSS v19.0 data package. Results were expressed as mean ± standard deviation; P < 0.05 was considered statistically significant.

## Results

### Biochemical Index Analysis

The changes of body weight, kidney–body ratio, SCr, and BUN content after 21 days of rat modeling are shown in **Table S1**. The body weight of the rats in the model group and the treatment group on the 21st day are significantly lower than those in the control group (p < 0.05), and the treatment group has an increasing trend compared with the control group, but there is no significant difference (p > 0.05). The kidney–body ratio is significantly higher than those in the control group (p < 0.05), and the treatment group has a reduced trend compared with the control group, but there is no significant difference (p > 0.05).The serum of rats was taken and the SCr and BUN contents were determined by a full-automatic biochemical analyzer (model KONELAB301). The results showed that the SCr and BUN contents in the model group and the treatment group were significantly higher than those in the control group. Compared with the model group, the contents of SCr and BUN in the drug administration group were both decreased, and were recalled to the control group, but there was no significant difference in SCr, and BUN showed significant difference (p < 0.01). This indicates that the rat model was successfully replicated.

### Methodological Verification

Before the formal analysis of serum samples, the conditions of UPLC-Q-TOF-MS/MS system were investigated for methodology. Forty microliters of each sample was extracted and mixed evenly to prepare quality control (QC) samples. The same QC sample was injected repeatedly for six times in succession. The results showed that the RSD% of the retention time and peak area of precision were lower than 0.25% and 3.56% respectively. QC solutions of six different samples were prepared by the same preparation method and injected into the detection system. The results showed that RSD% of retention time and peak area was lower than 0.14% and 3.79%, respectively. By comparing the six QC samples kept for 24 h in the autosampler (4°C) with the six same QC samples newly prepared, the stability of the samples was tested, and the RSD% of retention time and peak area were lower than 0.78% and 5.40%, respectively. According to the above, this method meets the requirements of this study. During the whole experiment, one QC sample was run in the middle of every 10 samples to maintain the stability of the system.

### Serum Metabolic Profile

**Figure 1:**
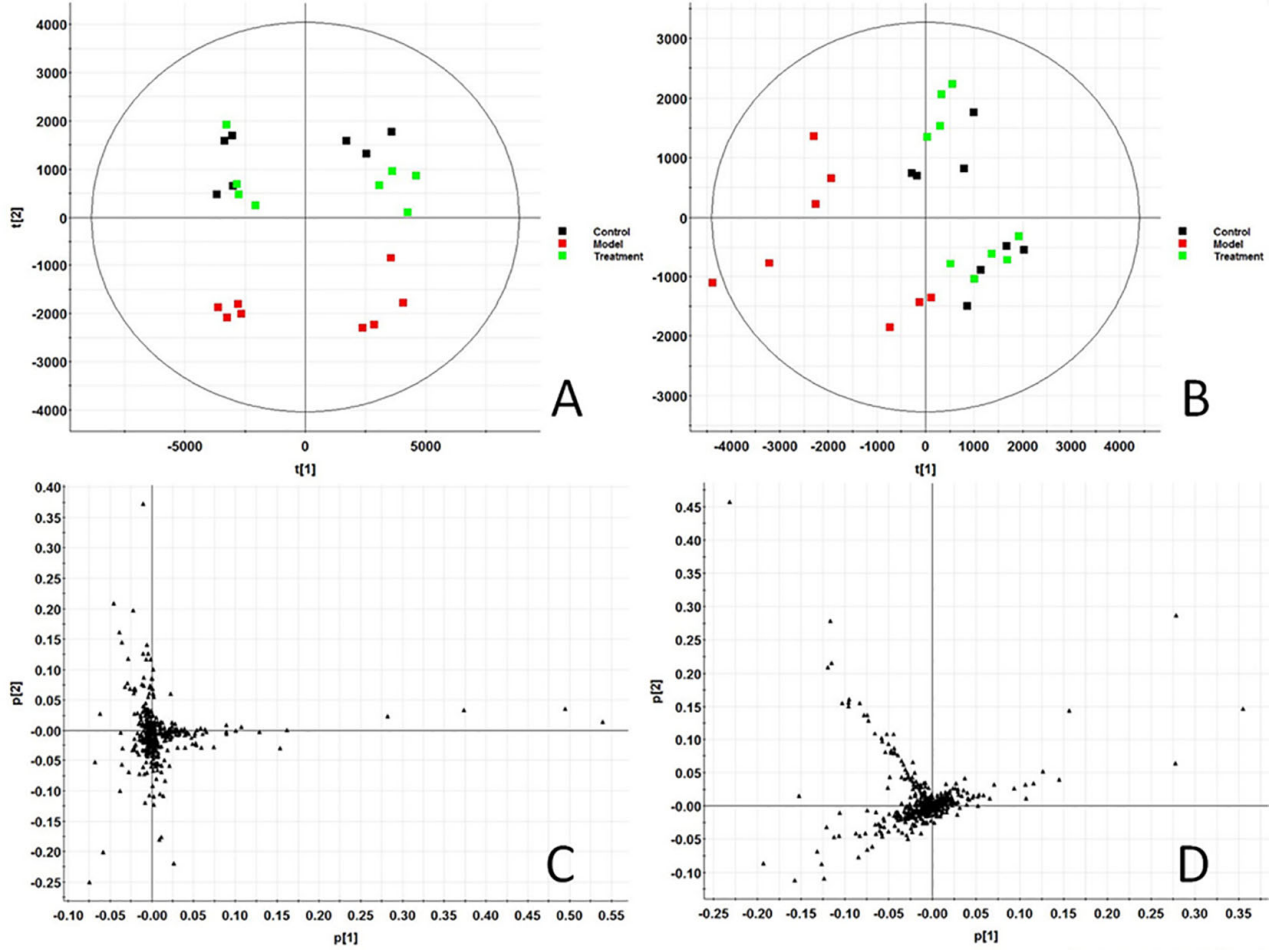
Score plot of serum profile of Control group, model group and treatment group scanned by PCA analysis. **(A)** PCA score in positive ion mode. **(B)** PCA score in negative ion mode. **(C)** Loading score in positive ion mode. **(D)** Loading score in negative ion mode.

In this study, all serum samples were scanned in ESI+ and ESI− ion mode under the stable UPLC-Q-TOF-MS/MS system. MassLynx V4.1 software (Waters, USA) integrates data analyzed by UPLC-MS/MS platform to obtain representative BPI metabolic profiles (**Figures S1A–C** and **Figures S2A–C**) in different groups of samples. It was found that the serum metabolic profile expression of each group was similar, but there were obvious content differences in many places. In order to further find metabolites with different content expressions, this study conducted non-targeted metabolomics analysis on renal fibrosis model rats based on UPLC-MS/MS technology, and imported all the original data into Progenesis QI software for peak alignment, peak extraction, and normalization. Then the ion information was transferred to Ezinfo3.0.3 software for multivariate data analysis. The PCA score chart (**Figure 1**) of PCA showed that the metabolic profiles in each group were clustered clearly and separated obviously; the treatment group was also far away from the model group and approached to the control group. 3D plot (**Figures S1D** and **S2D**) could also clearly observe the obvious spatial separation of each group, indicating that the serum metabolic level of the model rats had obvious changes and showed certain effects after AP treatment. This indicates that the rat model of renal fibrosis has been successfully replicated at the level of serum metabolism.

### Screening and Identification of Potential Biomarkers

**Figure 2:**
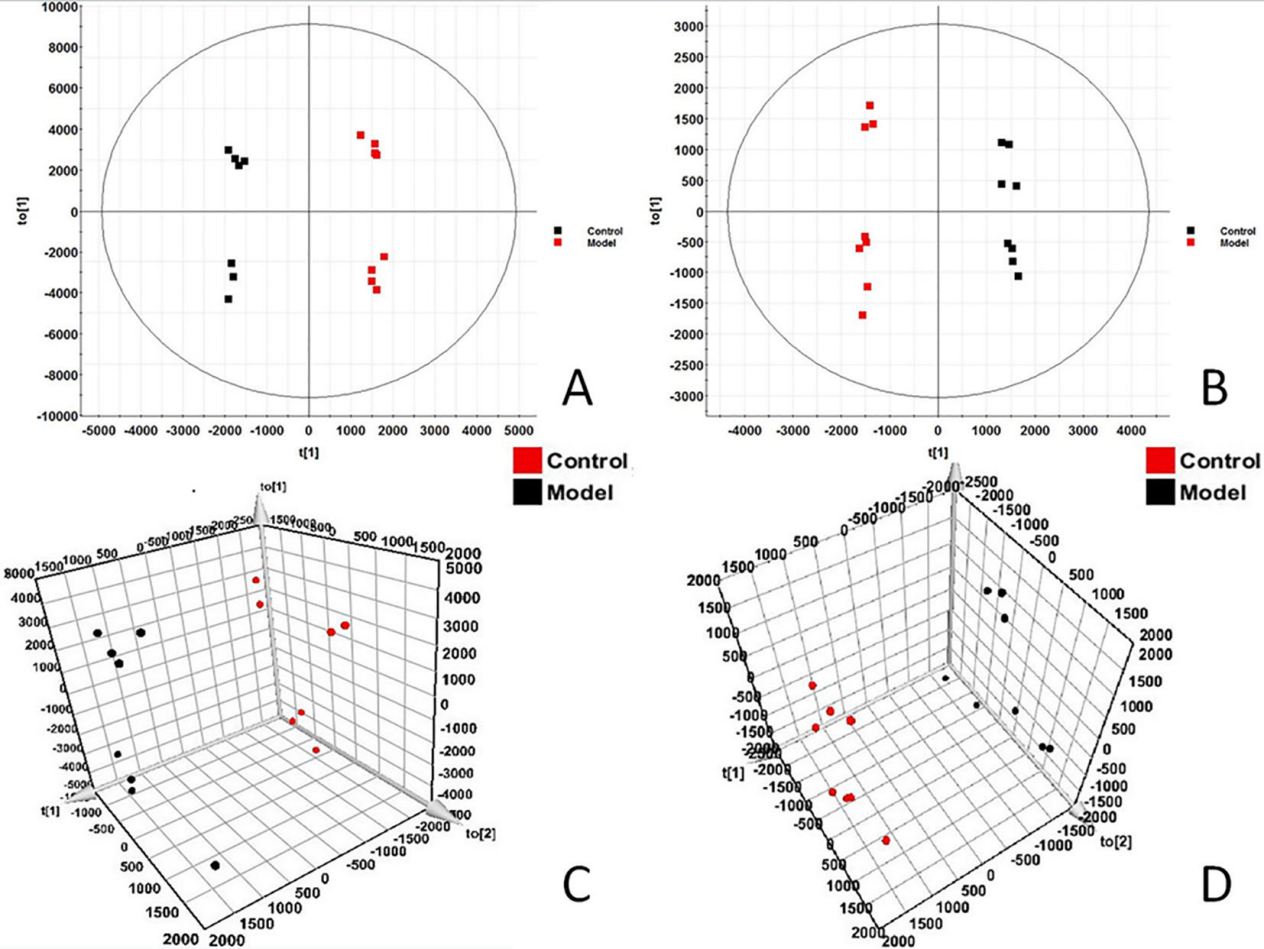
OPLS-DAscore and 3D-score plot of serum profile of control group and model group scanned by OPLS-DA analysis. **(A)** OPLS-DAscore inpositive ion mode. **(B)** OPLS-DAscore innegative ion mode. **(C)** 3D-Plotscore inpositive ion mode. **(D)** 3D-Plotscore in negative ion mode.

**Figure 3:**
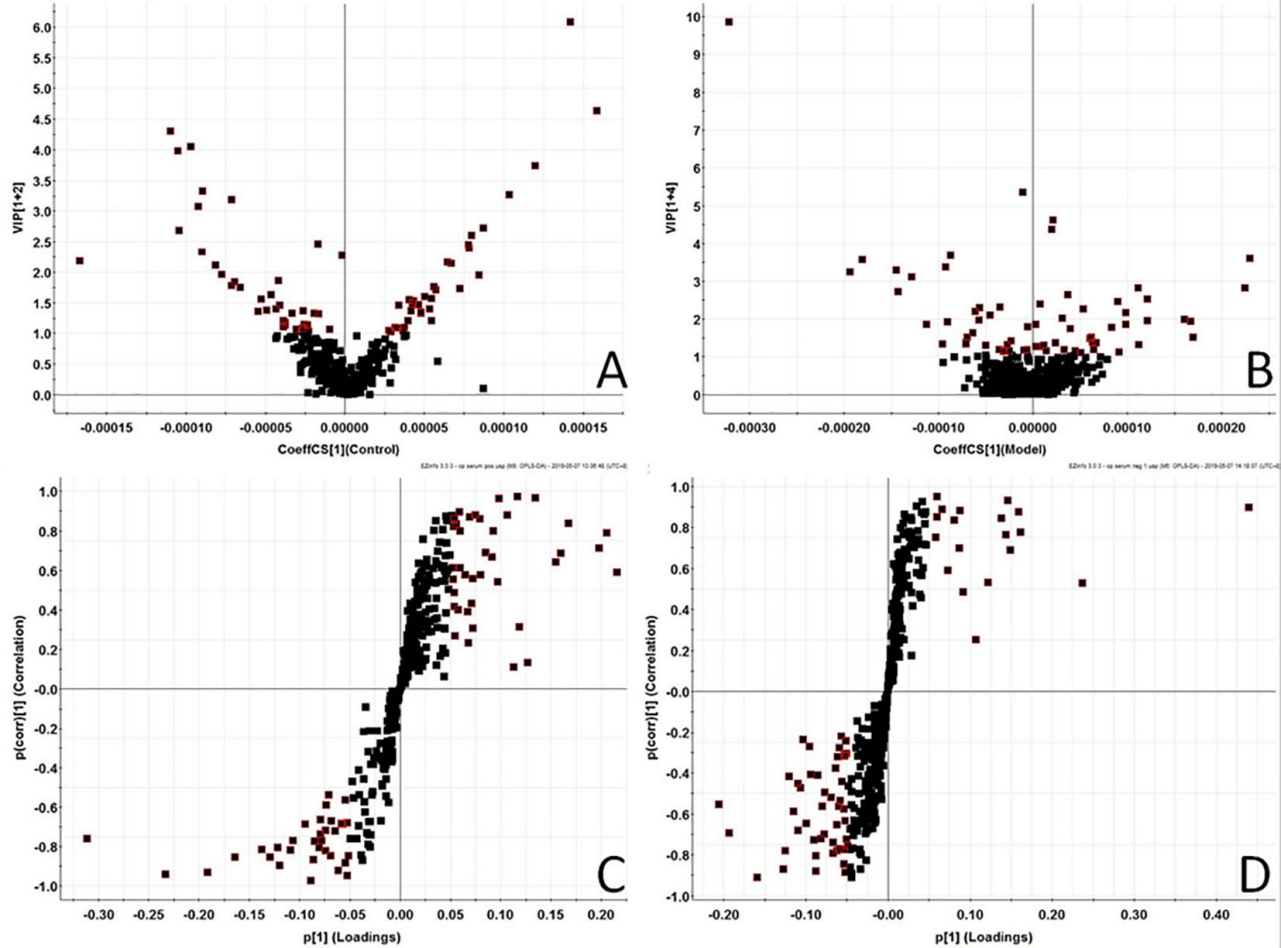
Metabolomics profiling of the control group and model group. VIP-plot of serum profile of control group and model group scanned by OPLS-DA analysis inpositive ion mode. **(B)** VIP-plot of serum profile of control group and model group scanned by OPLS-DA analysis innegative ion mode. **(C)** S-plot of serum profile of control group and model group scanned by OPLS-DA analysis in positive ion mode. **(D)** S-plot of serum profile of control group and model group scanned by OPLS-DA analysis in negative ion mode.

**Figure 4:**
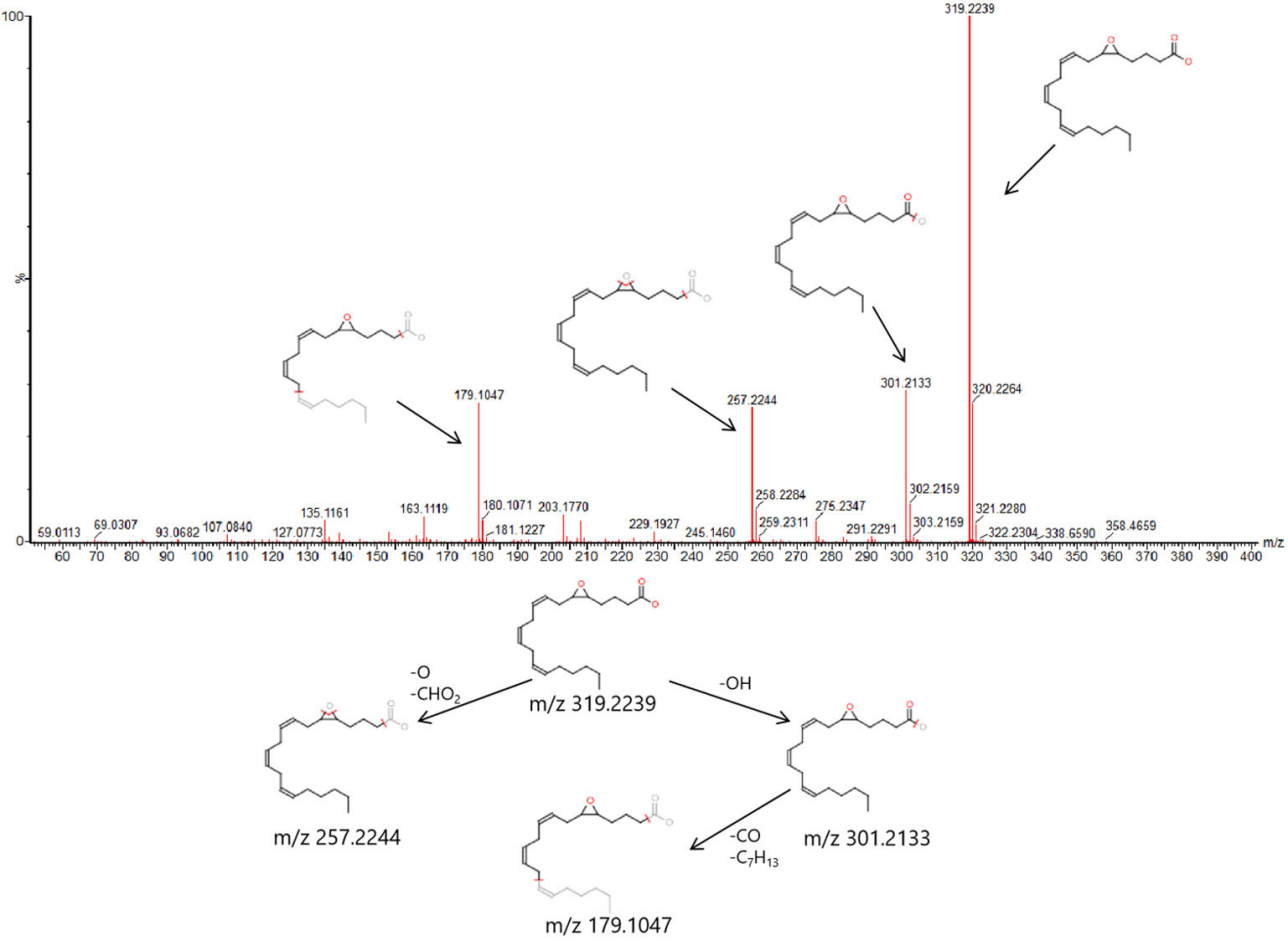
6.91-319.2239 (tR-m/z) compound secondary mass spectrometry fragment attribution and cracking process based on UPLC-Q-TOF-MS/MS .

In order to find out the different metabolites that affect the metabolic profile changes, the serum metabolic profile data of rats in the control group and the model group were further analyzed by OPLS-DA, and OPLS-DA plot (**Figures 2A, B**) and 3D plot (**Figures 2C, D**) between the control group and the model group were obtained. The Figure shows that the two groups are significantly separated. Furthermore, VIP plot diagrams (**Figures 3A, B**) and s-plot diagrams (**Figures 3C, D**) which can reflect the contribution rate between groups are obtained. In VIP plot, the farther ions are from the origin, the greater the contribution to the difference between the two groups. Carry out independent sample T-Test on the obtained data, screen potential endogenous biomarkers with p < 0.05 and VIP value greater than 1, and provide information including retention time, accurate quality, and MS/MS data according to the UPLC-MS platform. The precise molecular weight of the compound was determined by Q-TOF method within a reasonable measurement error range, and the element composition, unsaturation, and other information of the compound were obtained. By referring to literatures and data retrieval platforms such as HMDB and KEGG, the structure of potential endogenous marker MS/MS data is analyzed to determine the possible chemical structure. Combining the secondary fragment information provided by Masslynx V4.1, matching is carried out to obtain accurate structure and cracking information. Taking 6.91-319.2239 5,6-epoxy-8,11,14-eicosanoic acid as an example, the matching and cracking rules of secondary fragment information are shown in **Figure 4**. Finally, we identified and characterized 32 potential biomarkers, 16 in positive and 16 in negative ion mode. See **Table S2** for specific information. Compared with the control group, the 17 metabolite content expression in the model group was significantly increased. The levels of 15 metabolites were significantly decreased. Among them, 25 biomarkers showed extremely significant differences.

### Related Metabolic Pathways

**Figure 5:**
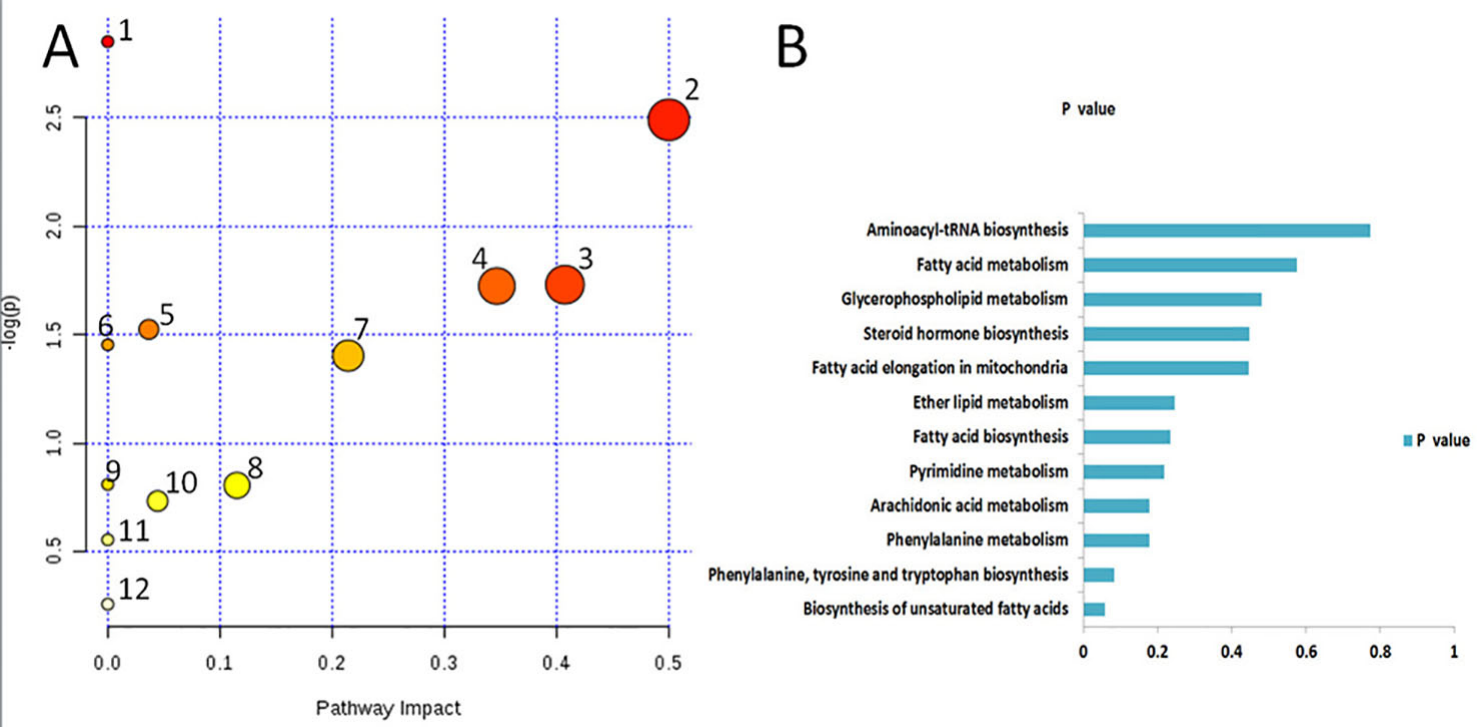
Metabolic pathways associated with potential biomarkers in rat serum. **(A)** The map was generated using MetaboAnalyst. 1. Biosynthesis of unsaturated fatty acids. 2. Phenylalanine, tyrosine and tryptophan biosynthesis. 3. Phenylalanine metabolism. 4. Arachidonic acid metabolism. 5. Pyrimidine metabolism. 6. Fatty acid biosynthesis. 7. Ether lipid metabolism. 8. Fatty acid elongation in mitochondria. 9. Steroid hormone biosynthesis. 10. Glycerophospholipid metabolism. 11. Fatty acid metabolism. 12. Aminoacyl-tRNA biosynthesis. **(B)** P values of 12relatedmetabolic pathways.

**Figure 6:**
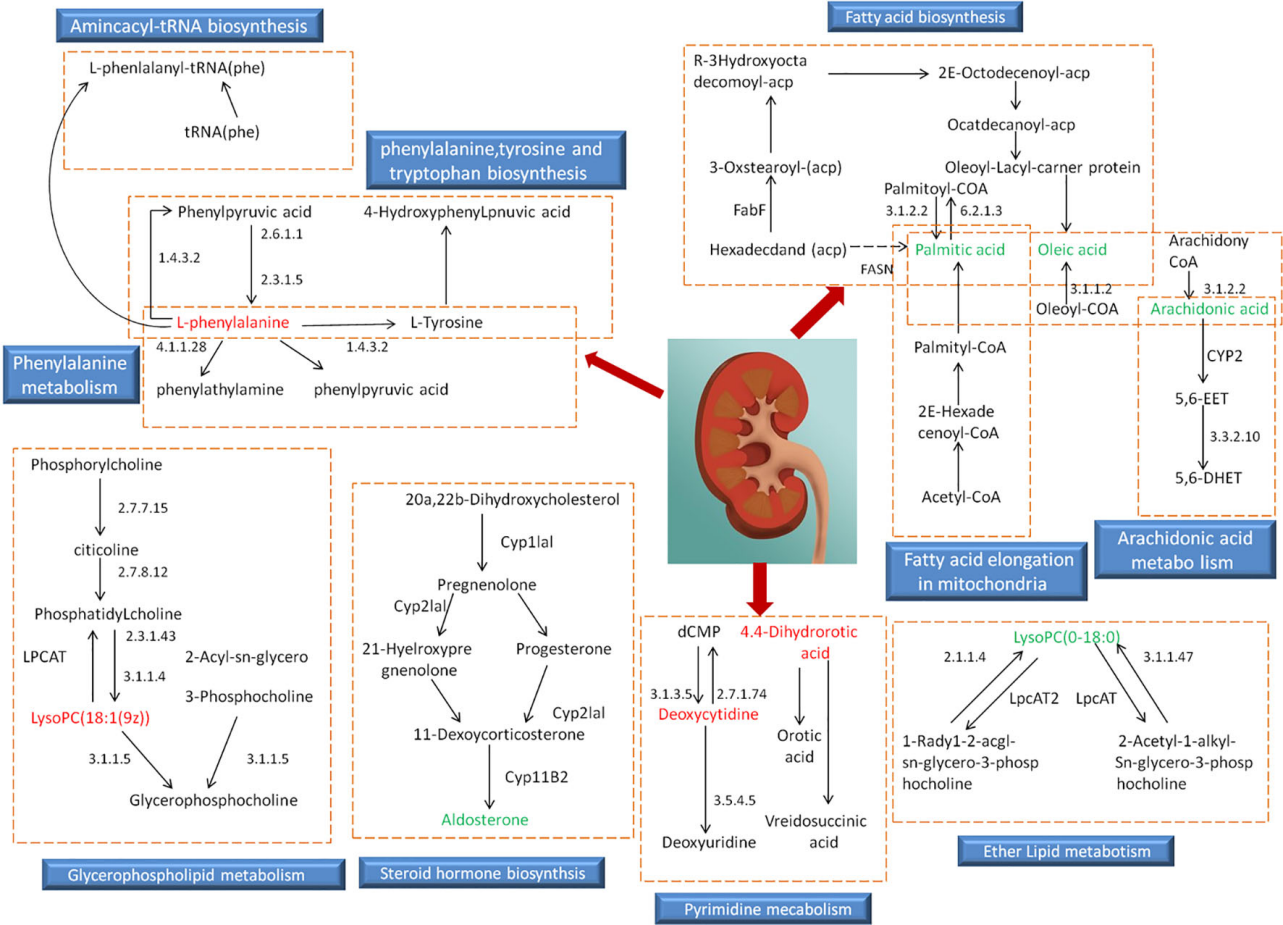
Relevant network diagram of potential biomarkers related to renal fibrosis model based on KEGG. Red is the potential biomarker of high expression after modeling, green is the potential biomarker of low expression, metabolites in orange frame belong to the same metabolic pathway, and blue is the name of the relevant metabolic pathway.

Based on the identified endogenous biomarkers, MetaboAnalyst and KEGG and other network databases were used for enrichment analysis of relevant metabolic pathways to explore the metabolic pathways affected in serum metabolism of renal fibrosis rats (**Figure 5**). Twelve metabolic pathways including biosynthesis of unsaturated fatty acids were characterized. Phenylalanine, tyrosine, and tryptophan biosynthesis, phenylalanine metabolism, arachidonic acid metabolism, pyrimidine metabolism, fatty acid biosynthesis, ether lipid metabolism, fatty acid elongation in mitochondria, steroid hormone biosynthesis, glycerophospholipid metabolism, fatty acid metabolism, aminoacyl-tRNA biosynthesis. Among them, phenylalanine, tyrosine, and tryptophan biosynthesis, phenylalanine metabolism, and arachidonic acid metabolism pathway impact is greater than 0.3. It may be the most important metabolic pathway in the pathogenesis and development of renal fibrosis model rats. **Figure 6** is a correlation network diagram of potential biomarkers related to renal fibrosis models based on KEGG network. In the Figure, red is a potential biomarker with high expression after modeling, green is a potential biomarker with low expression, metabolites in the orange box belong to the same metabolic pathway, and blue is the name of the relevant metabolic pathway; it can be seen that the metabolic pathway in rats changes after the model is established.

### Effect of AP on Metabolic Profile

**Figure 7:**
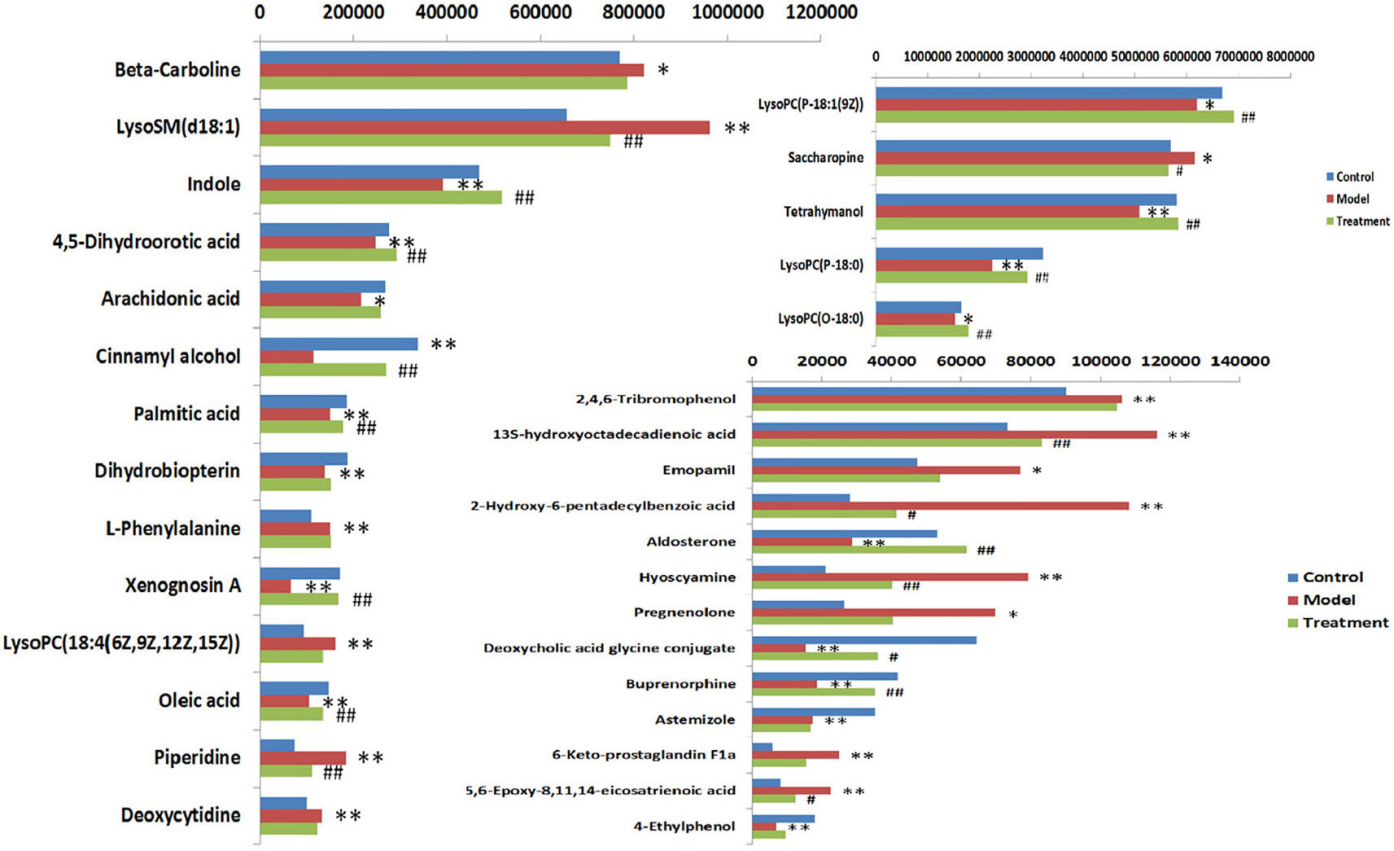
Heat map analysis of 32 differential metabolites in control group, model group and treatment group rats. Different color marks represent the change degree of relative content and the darker the color, the higher the relative content. Each row represents a metabolite, and each column represents a single sample. *model vs control, p < 0.05; **model vs control, p < 0.01. ^#^Treament vs model, p < 0.05; ^##^Treament vs model, p < 0.01.

**Figure 8:**
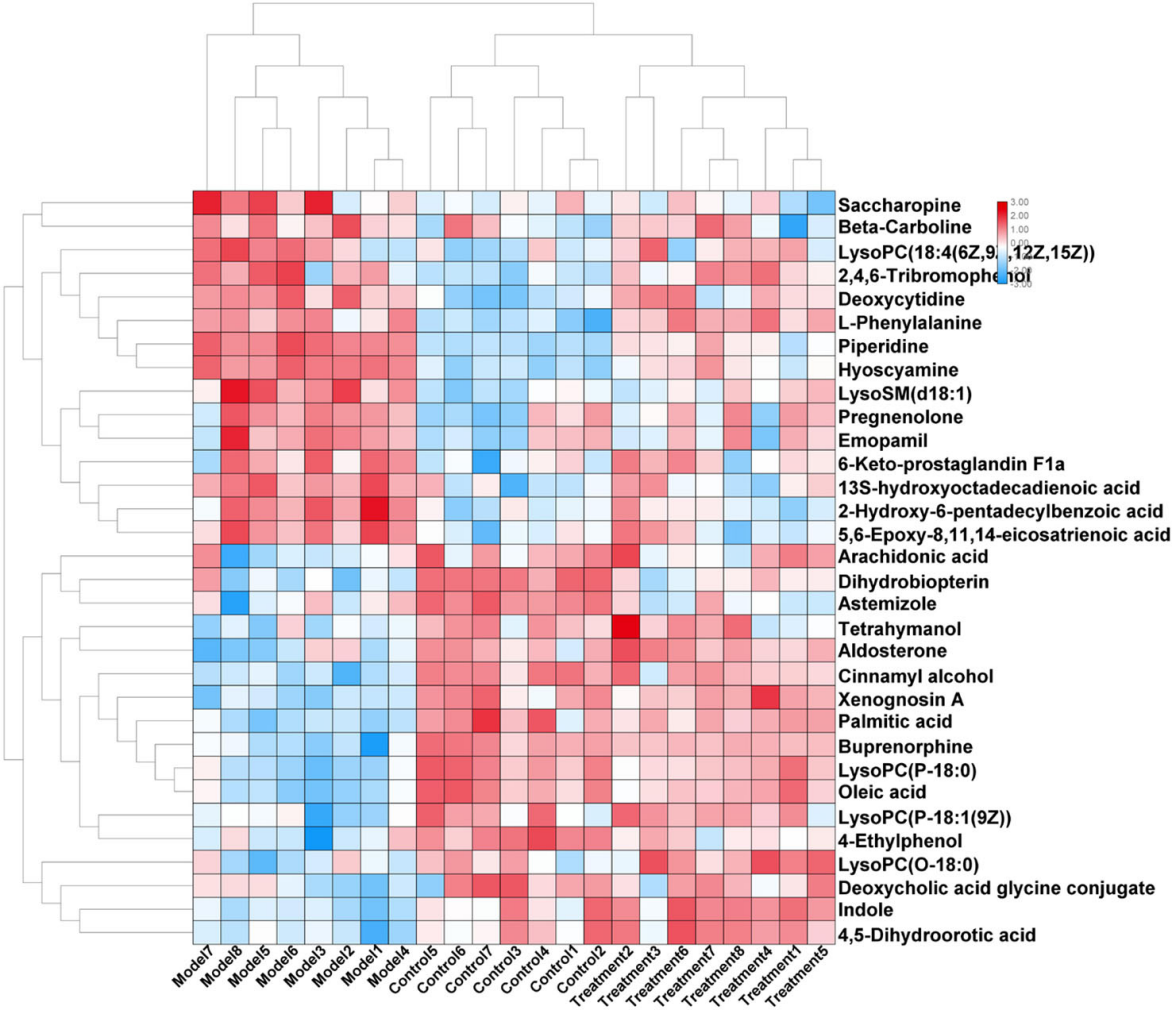
Changes in serum relative content of potential biomarkers after AP treament. The depth of the color represents the change of the content of the corresponding marker.

In order to evaluate the effect of AP on metabolic profile of renal fibrosis rat model, PCA was carried out on the blood metabolic profile of three groups of rats, the results manifest the blood metabolism spectrum of the treatment group was far away from the model group and close to the control group. The results manifest AP can significantly reverse the metabolic profile of renal fibrosis model rats and make them approach a healthy state, further indicating that AP plays a certain intervention role in the occurrence and development of renal fibrosis model rats. In addition, by analyzing the change trend of potential biomarker content of AP on renal fibrosis model rats, it is found that AP intervention can affect the content of potential biomarkers and make them regulate to the direction of control group. The specific information is shown in **Table S2**. And in the bar graph (**Figure 7**), we can see that among the 32 potential biomarkers identified, compared with model group, AP treatment group can regulate 30, of which 4 have significant difference (p < 0.05) and 16 have extremely significant differences (p < 0.01). In order to further study the differences of potential biomarkers between different groups, cluster thermal analysis of 32 metabolites, such as **Figure 8**, reveals the changes in the relative content of potential biomarkers between the two groups. Studies on biomarkers of AP callback show that its main metabolic pathways involved in regulation are phenylalanine, tyrosine and tryptophan biosynthesis; phenylalanine metabolism; arachidonic acid metabolism; glycerophospholipid metabolism, etc. This indicates that AP may play a role in preventing and treating renal fibrosis by interfering with the above metabolic pathways.**

## Discussion

Renal fibrosis is a pathological repair reaction of kidney to chronic injury. It is characterized by excessive proliferation and deposition of extracellular matrix components in kidney which is the common pathological basis of various chronic kidney diseases and an important way for chronic kidney diseases to progress to end-stage renal failure. Therefore, early prevention or even reversal of renal fibrosis is of great significance for the prevention and treatment of end-stage renal failure. TGF-β has been the focus of attention in renal fibrosis and anti-renal fibrosis treatment (Wang et al., 2012). Renal fibrosis caused by TGF-β1 is mainly manifested in the following aspects: it can stimulate fibroblasts and other factors to increase ECM component synthesis; by inhibiting the activities of various ECM degrading enzymes (MMPs) and plasminogen activator, and stimulating the activities of tissue inhibitor of metalloproteinase-1 (TIMPS) and plasminogen activator-1 (PAI-1), ECM degradation is inhibited. Stimulating renal tubular epithelial cells to transdifferentiate into MFBs; increase the expression of ECM receptors such as integrins, thus increasing the interaction between ECM and cells, etc. Overexpression of collagen is an important link in the formation of renal interstitial fibrosis, and TGF-β1 is the most important regulator of type I collagen (Jiang et al., 2016). Anti-TGF-β is considered as one of the promising methods for anti-fibrosis treatment (Doi et al., 2011). However, the pathological mechanism of organ fibrosis is complex, and there are often multiple links and targets. The intervention effect of a single target may not be good (Zeisberg et al., 2002). However, TCM has the characteristics of multi-component and multi-link action, and often has comprehensive advantages in diseases with complicated pathological changes such as renal fibrosis. In this study, a rat model of renal fibrosis was established by unilateral ureteral ligation. The serum of control group, model group, and AP treatment group were analyzed based on high-throughput metabolomics combined with quadrupole time-of-flight mass spectrometry technology. The differences of metabolic profiles of endogenous metabolites in serum of rats between different groups were studied. The results showed that on the 21st day of model replication, the model group and control group were clustered obviously. Through searching HMDB, MetPA, KEGG, and other databases, 32 potential biomarkers that have significant impact on clustering grouping were finally locked, 16 in positive and negative ion mode respectively, and a total of 12 related metabolic pathways were characterized by metabolic pathway enrichment analysis, of which the most relevant are phenylalanine, tyrosine, and tryptophan biosynthesis; phenylalanine metabolism; and arachidonic acid metabolism, which indicates that these metabolic pathways may be the pathological mechanism in the process of renal fibrosis. After 21 days of gastric administration of AP solution, 30 potential biomarkers can be recalled, of which 20 have significant differences, which indicates that AP can achieve the effect of treating renal fibrosis by adjusting the metabolic level of these markers. The metabolic pathways most related to this change are arachidonic acid metabolism and glycerophospholipid metabolism.

Previous studies have shown that AP has a significant effect on the treatment of kidney diseases (Jiang et al., 2016). Studies have found that APS can effectively reduce diabetic glomerular fibrosis induced by high fat plus low dose STZ in rats and inhibit the overexpression of TGF-β1 in damaged renal tubular epithelial cells (Zhang et al., 2007). In the kidney yang deficiency diabetic rat model, AP can also inhibit the overexpression of TGF-β1 mRNA, thus delaying the progress of diabetic nephropathy. Some studies have confirmed that renal tissue fibrosis can occur in rats with left renal vein stenosis. AM can reduce renal tissue fibrosis damage by reducing the expression of TGF-β1 (Lan, 2011; Qin et al., 2011). In this study, AP interferes with the content and activity of serum metabolites in rats with renal fibrosis induced by left ureter ligation. It can be seen that the changes of serum metabolites in rats with AP interference are mainly related to renal tubular fibrosis, glomerular mesangial cell accumulation, inflammation, oxidative stress, lipid metabolism, etc. Among the 32 metabolic biomarkers characterized by the model group, 2 important substances: arachidonic acid and 5-EET are involved in arachidonic acid metabolism; involved in unsaturated fatty acid metabolism are palmitic acid, oleic acid, and arachidonic acid; glycerol phospholipid metabolism: LysoPC [18:1(9z)]; biosynthesis of steroid hormone matches pregnenolone, aldosterone; phenylalanine metabolic pathway: L-phenylalanine.

Fatty acid biosynthetic pathways include palmitic acid, oleic acid, and arachidonic acid; palmitic acid is a free fatty acid component, a donor of cell membrane lipid structure and prostaglandin synthesis, and an intermediate product of fatty acid metabolism. It participates in the β-oxidation process of fatty acid and plays a role in energy metabolism and fatty acid digestion (Lu et al., 2003). Oleic acid is a monounsaturated ω-9 fatty acid, which is the most widely distributed fatty acid with the highest fat content in nature. Both can induce apoptosis of interstitial cells by producing ceramide (Mu et al., 2001). Studies have shown that palmitic acid can induce apoptosis of renal tubular epithelial cells, upregulate cPLA2, produce bioactive components such as free fatty acid and lysolecithin, which are mainly arachidonic acid, and participate in the occurrence and development of tissue fibrosis (Baggio et al., 2005; Allison, 2015).

LysoPCs is a lysophosphate compound, which can promote epithelial cell apoptosis, increase vascular permeability, promote fibroblast migration and anti-apoptosis ability, activate the activity of potential TGF-β1, increase the secretion of fibrogenic factors PDGF-β and CTGF by proximal tubule cells, mediate the role of lysophosphatidic acid and sphingosine 1-phosphate in fibrosis, and accelerate the process of fibrosis. In addition, LysoPC can promote oxidative stress, inhibit glucose transport and insulin-mediated glucose metabolism, and inhibit the activity of Na^+^–K^+^–ATP enzyme, thus causing abnormalities in cell structure and function. The changes of LysoPCs are closely related to the renal fibrosis process and oxidative stress reaction in rats with renal fibrosis (Kang et al., 2015; Zeng et al., 2017; Sun et al., 2018;).

The metabolic pathway of arachidonic acid includes 5,6-epoxy-8,11,14-eicosanoic acid; arachidonic acid has two kinds of metabolism, among which arachidonic acid has the effects of esterifying cholesterol, increasing vascular elasticity, reducing vascular viscosity, regulating blood cell function, etc. Peanut-like acids, such as 20-HETE and EETs, which have the function of regulating the transport of renal tubular epithelial cells, can be produced by cytochrome P450 cyclooxygenase, which is of great significance for maintaining renal function of patients with nephropathy (Maier and Roman, 2001). In addition, arachidonic acid is the direct premise of prostaglandins, leukotrienes, and other substances, and has important regulatory effects on lipid protein metabolism, vascular rheology, vascular elasticity, inflammatory response, and so on (Skibba et al., 2017). 5,6-epoxy-8,11,14-eicosartienoic acid is a metabolite produced by arachidonic acid through cytochrome P450 cyclooxygenase, and the changes of the contents of the two are closely related to the inflammatory reaction, high blood viscosity, and the characterization of dyslipidemia in renal fibrosis rats (Xiang et al., 2016). The influence of these pathways explains from a certain angle that renal fibrosis may be a comprehensive disease, and it is also an explanation that renal fibrosis is difficult to be completely controlled by a single drug. However, the complex and diverse components of TCM may realize multi-target synergistic effect and treat comprehensive kidney diseases.

Metabolomics is an important branch of system biology. It mainly studies the changes of all metabolites produced by external stimuli. Metabolomics takes metabolic product groups in organisms as analysis targets, adopts high-throughput detection as technical means (chromatography, mass spectrometry, nuclear magnetic resonance, spectrum, etc.) (Wang et al., 2019), uses data processing for systematic integration, combines qualitative detection technology and quantitative characterization methods to discover possible small molecular compounds, interprets the biological significance contained in the data by means of bioinformatics platform, and excavates potential biomarkers (Zhang et al., 2019). So as to further explore the internal metabolic circulation pathway and signal pathway of the disease and provide possibility for exploring the development process and mechanism of the disease (Sun et al., 2015).

The occurrence and development of renal diseases involve changes in the expression levels of many functional genes and proteins, and minor changes in gene and protein expression can be amplified in small molecule metabolism, so high-throughput metabolomics can be used for accurate identification. Therefore, this study is based on the research method of high-throughput metabolomics. With the help of UPLC-Q-TOF/MS and other advanced instruments and equipment, the blood of renal fibrosis model rats established by unilateral tubal ligation operation is analyzed, and the metabolic fingerprint of serum of renal fibrosis rats is established. Combined with PCA and OPLS-DA analysis, potential biomarkers and metabolic pathways related to renal fibrosis are found, and 32 biomarkers and 12 metabolic pathways related to renal fibrosis are finally determined. It is also found that 20 potential biomarkers can be recalled after AP treatment, and there is a statistical difference compared with the model group. These biomarkers mainly involve changes in metabolic pathways such as arachidonic acid metabolism and glycerophospholipid metabolism, thus providing a basis for exploring the pathogenesis of renal fibrosis model rats and the therapeutic effect of AP. This study is the first time to explain the preventive and therapeutic effects of oral AP on renal fibrosis from the perspective of metabolic pathway, and to prove the effectiveness and time potential of metabolomics in the study of intervention effects of TCM.

## Conclusion

In this study, a rat model of renal fibrosis was established by unilateral tubal ligation. Based on UPLC-Q-TOF/MS and multivariate data analysis, 32 potential biomarkers related to renal fibrosis and 12 related metabolic pathways were revealed from the level of serum metabolomics. AP can recall 30 potential markers, 16 of which have significant difference (p < 0.01). Compared with the model group, four have significant differences, mainly regulating three metabolic pathways. Furthermore, it will affect the metabolic level of renal fibrosis model rats and make them develop towards remission, thus providing support for further research on AP treatment of renal fibrosis.

## Data Availability Statement

The raw data supporting the conclusions of this article will be made available by the authors, without undue reservation, to any qualified researcher.

## Ethics Statement

The animal study was reviewed and approved by Guilin Medical University.

## Author Contributions

LR conceived and designed the experiments. LR, X-YG, FG, M-LJ, and X-NS performed the experiment. X-YG analyzed the data. FG guided the experiment. LR wrote the paper. All authors read and approved the final manuscript.

## Conflict of Interest

The authors declare that the research was conducted in the absence of any commercial or financial relationships that could be construed as a potential conflict of interest.
