# Supplementary material for: Identification of the Perturbed Metabolic Pathways Associating With Renal Fibrosis and Evaluating Metabolome Changes of Pretreatment With Astragalus polysaccharide Through Liquid Chromatography Quadrupole Time-Of-Flight Mass Spectrometry
Source: Front Pharmacol. 2020 Jan 29;10:1623. doi: 10.3389/fphar.2019.01623 (PMC7000425; doi:10.3389/fphar.2019.01623)
Supplement: Supplementary file 1 [file DataSheet_1.doc]

Table S1：Table of changes in body weight, kidney-body ratio, SCr, BUN content

| Group | Weight | kidney-body ratio | SCr(μmol/L) | BUN(μmol/L) |
| --- | --- | --- | --- | --- |
| Control | 325±10.15 | 0.79±0.35 | 19.56±2.36 | 8.24±0.61 |
| Model | 294.3±9.21* | 4.53±2.36* | 35.98±5.24** | 33.47±2.59** |
| Treatment | 301.6±9.56 | 3.01±1.25 | 29.12±3.21* | 25.6±2.01** |

Table S2：The detailed information of potential biomarkers

| Rt/min-M/Z | Exact mass | Scan mode | M+X | Proposed composition | Predicting compound | HMDB ID | KEGG ID | Mass Error (ppm) | Mass fragment | Chang trend | trend | Chart |
| --- | --- | --- | --- | --- | --- | --- | --- | --- | --- | --- | --- | --- |
| 11.21_429.4082 | 428.4018 | ESI+ | M+H | C_3_0H_52_O | Tetrahymanol | HMDB0006836 | C06083 | -1.97 | 429[M+H]^+^,411[M+H-H_2_O]^+^,355[M+H-C_4_H_10_O]^+^ | ↓** | ↑## | 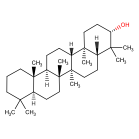 |
| 12.58_299.1202 | 276.1321 | ESI+ | M+Na | C_11_H_2_0N_2_O_6_ | Saccharopine | HMDB0000279 | C00449 | -4.20 | 277[M+H]^+^,241[M+H-H_3_O_2_]^+^,231[M+H-CH_3_O_4_]^+^,185[M+H-C_2_H_3_O_4_]^+^ | ↑* | ↓# | 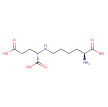 |
| 9.54_466.3514 | 465.3457 | ESI+ | M+H | C_23_H_5_0N_2_O_5_P+ | LysoSM(d18:1) | HMDB0006482 | C03640 | -3.54 | 466[M+H]^+^ | ↑** | ↑## | 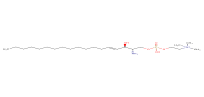 |
| 2.35_157.0629 | 134.0732 | ESI+ | M+Na | C_9_H_10_O | Cinnamyl alcohol | HMDB0029697 | C02394 | 3.63 | 157[M+Na]^+^,117[M+H-OH]^+^,103[M+H-CH_3_O]^+^ | ↓** | ↓## | 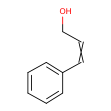 |
| 2.22_86.0966 | 85.15 | ESI+ | M+H | C_5_H_11_N | Piperidine | HMDB0034301 | C01749 | 1.98 | 86[M+H]^+^,69[M+H_3_N]^+^ | ↑** | ↑## | 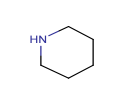 |
| 2.22_290.1723 | 289.1678 | ESI+ | M+H | C_17_H_23_NO_3_ | Hyoscyamine | HMDB0014568 | C02046 | -9.41 | 290[M+H]^+^,149[M+H-C_8_H_14_NO]^+^,124[M+H-C_9_H_9_O_3_]^+^ | ↑** | ↑## | 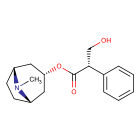 |
| 1.95_118.0642 | 117.0578 | ESI+ | M+H | C_8_H_7_N | Indole | HMDB0000738 | C00463 | -7.50 | 118[M+H]^+^,92[M+H-C_2_H]^+^,90[M+H-C_2_H_3_]^+^ | ↓** | ↑## | 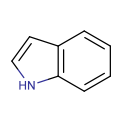 |
| 6.49_240.1112 | 239.1018 | ESI+ | M+H | C_9_H_13_N_5_O_3_ | Dihydrobiopterin | HMDB0000038 | C02953 | 8.54 | 240[M+H]^+^,164[M+H-C_3_H_6_O]^+^,121[M+H-C_4_H_7_NO_2_]^+^,112[M+H-C_5_H_6_N_2_O]^+^ | ↓** | ↓ | 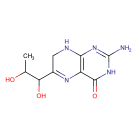 |
| 10.31_305.2504 | 304.2402 | ESI+ | M+H | C_20_H_32_O_2_ | Arachidonic acid | HMDB0001043 | C00219 | 9.56 | 305[M+H]^+^,287[M+H-OH]^+^,269[M+H-H_3_O]^+^,259[M+H-CHO_2_]^+^,245[M+H-C_2_H_3_O_2_]^+^ | ↓* | ↓ | 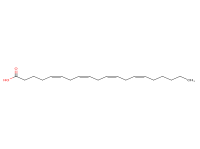 |
| 0.76_191.0596 | 168.0687 | ESI+ | M+Na | C_11_H_8_N_2_ | Beta-Carboline | HMDB0012897 | C20157 | 9.61 | 191[M+Na]^+^,169[M+H]^+^,142[M+H-CN]^+^,116[M+H-C_3_H_2_N]^+^ | ↑* | ↑ | 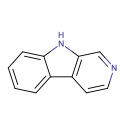 |
| 0.84_250.0799 | 227.0906 | ESI+ | M+Na | C_9_H_13_N_3_O_4_ | Deoxycytidine | HMDB0000014 | C00881 | 0.24 | 250[M+Na]^+^,228[M+H]^+^,112[M+H-C_5_H_10_NO_3_]^+^ | ↑** | ↑ | 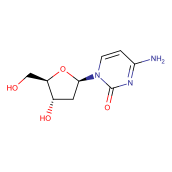 |
| 9.35_317.2469 | 316.2402 | ESI+ | M+H | C_21_H_32_O_2_ | Pregnenolone | HMDB0000253 | C01953 | -1.84 | 317[M+H]^+^,299[M+H-OH]^+^,273[M+H-C_2_H_3_O]^+^,259[M+H-C_3_H_5_O]^+^ | ↑* | ↑ | 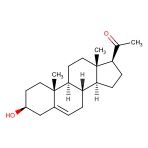 |
| 1.95_159.0392 | 158.0328 | ESI+ | M+H | C_5_H_6_N_2_O_4_ | 4,5-Dihydroorotic acid | HMDB0000528 | C00337 | -4.97 | 159[M+H]^+^,113[M+H-CHO_2_]^+^ | ↓** | ↑## | 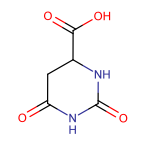 |
| 13.52_361.1984 | 360.1937 | ESI+ | M+H | C_21_H_28_O_5_ | Aldosterone | HMDB0000037 | C01780 | -7.00 | 367[M+H]^+^,343[M+H-OH]^+^,325[M+H-H_3_O_2_]^+^,311[M+H-CH_5_O_2_]^+^ | ↓** | ↑## | 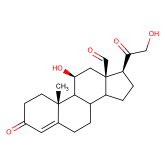 |
| 9.35_357.2299 | 334.2409 | ESI+ | M+Na | C_23_H_3_0N_2_ | Emopamil | HMDB0012224 | C13766 | -0.56 | 357[M+Na]^+^,335[M+H]^+^ | ↑* | ↑ | 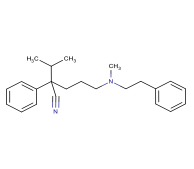 |
| 6.49_459.2516 | 458.2482 | ESI+ | M+H | C_28_H_31_FN_4_O | Astemizole | HMDB0014775 | C06832 | -8.38 | 459[M+H]^+^,439[M+H-F]^+^,427[M+H-CH_3_O]^+^,337[M+H-C_8_H_9_O]^+^ | ↓** | ↓ | 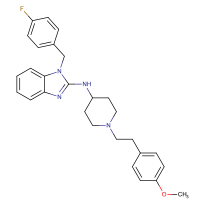 |
| 7.21_506.3645 | 507.693 | ESI- | M-H | C_26_H_54_NO_6_P | LysoPC(P-18:0) | HMDB0013122 | C04230 | 5.66 | 506[M-H]^-^,419[M-H-C_4_H_13_N]^-^,403[M-H-C_5_H_15_NO]^-^ | ↓** | ↓## | 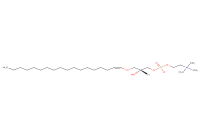 |
| 6.17_504.3483 | 505.677 | ESI- | M-H | C_26_H_52_NO_6_P | LysoPC(P-18:1(9Z)) | HMDB0010408 | C04230 | 4.70 | 504[M-H]^-^,417[M-H-C_5_H_3_N]^-^,265[M-H-C_8_H_18_NO_5_P]^-^ | ↓* | ↑## | 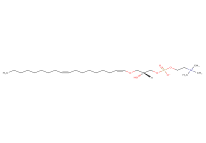 |
| 2.36_255.1034 | 256.1099 | ESI- | M-H | C_16_H_16_O_3_ | Xenognosin A | HMDB0029539 | C08731 | 2.74 | 255[M-H]^-^,239[M-H-CH_5_]^-^,223[M-H-CH_3_O]^-^ | ↓** | ↓## | 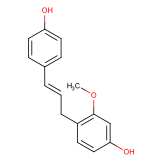 |
| 8.49_508.3770 | 509.709 | ESI- | M-H | C_26_H_56_NO_6_P | LysoPC(O-18:0) | HMDB0011149 | C04317 | -0.40 | 508[M-H]^-^,449[M-H-C_3_H_9_N]^-^ | ↓* | ↑## | 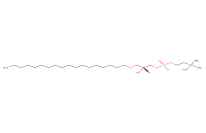 |
| 8.19_347.2609 | 348.2664 | ESI- | M-H | C_22_H_36_O_3_ | 2-Hydroxy-6-pentadecylbenzoic acid | HMDB0029683 | C10759 | 4.99 | 347[M-H]^-^,303[M-H-CHO_2_]^-^ | ↑** | ↑# | 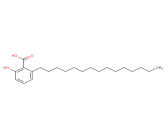 |
| 3.62_514.2977 | 515.628 | ESI- | M-H | C_26_H_46_NO_7_P | LysoPC(18:4(6Z,9Z,12Z,15Z)) | HMDB0010389 | C04230 | 7.37 | 514[M-H]^-^,275[M-H-C_8_H_18_NO_5_P]^-^ | ↑** | ↑ | 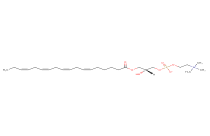 |
| 7.21_281.2472 | 282.2559 | ESI- | M-H | C_18_H_34_O_2_ | Oleic acid | HMDB0000207 | C00712 | -5.09 | 281[M-H]^-^,237[M-H-CHO_2_]^-^ | ↓** | ↓## | 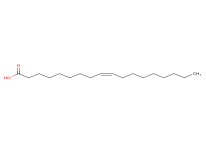 |
| 1.71_164.0719 | 165.079 | ESI- | M-H | C_9_H_11_NO_2_ | L-Phenylalanine | HMDB0000159 | C00079 | 1.10 | 164[M-H]^-^,147[M-H-NH_3_]^-^,103[M-H-CH_5_O_3_]^-^ | ↑** | ↑ | 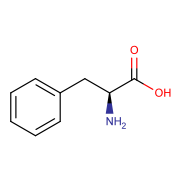 |
| 6.36_295.2269 | 296.2351 | ESI- | M-H | C_18_H_32_O_3_ | 13S-hydroxyoctadecadienoic acid | HMDB0004667 | C14762 | -3.31 | 295[M-H]^-^,277[M-H-H_3_O]^-^,233[M-H-CH_3_O_3_]^-^ | ↑** | ↑## | 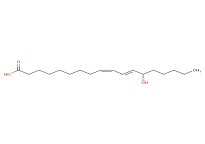 |
| 4.77_448.3088 | 449.3141 | ESI- | M-H | C_26_H_43_NO_5_ | Deoxycholic acid glycine conjugate | HMDB0000631 | C05464 | 4.38 | 448[M-H]^-^,386[M-H-CH_3_O_3_]^-^ | ↓** | ↓# | 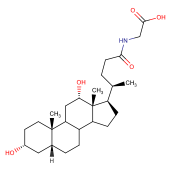 |
| 6.48_255.2344 | 256.43 | ESI- | M-H | C_16_H_32_O_2_ | Palmitic acid | HMDB0000220 | C00249 | 5.81 | 255[M-H]^-^,211[M-H-CHO_2_]^-^ | ↓** | ↓## | 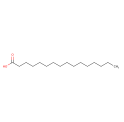 |
| 5.82_466.2971 | 467.3036 | ESI- | M-H | C_29_H_41_NO_4_ | Buprenorphine | HMDB0015057 | C08007 | 1.82 | 466[M-H]^-^,366[M-H-C_6_H_13_O]^-^ | ↓** | ↓## | 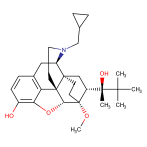 |
| 4.09_369.2265 | 370.2355 | ESI- | M-H | C_20_H_34_O_6_ | 6-Keto-prostaglandin F1a | HMDB0002886 | C05961 | -4.76 | 369[M-H]^-^,351[M-H-H_3_O]^-^,333[M-H-H_5_O_2_]^-^,325[M-H-CHO_2_]^-^ | ↑** | ↑ | 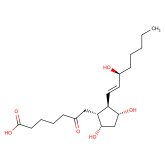 |
| 0.76_326.7674 | 327.7734 | ESI- | M-H | C_16_H_14_O_9_ | 2,4,6-Tribromophenol | HMDB0029642 | C14454 | 3.93 | 326[M-H]^-^,296[M-H-CH_3_Br_2_]^-^ | ↑** | ↑ | 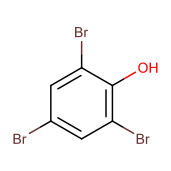 |
| 6.53_319.2284 | 320.2351 | ESI- | M-H | C_20_H_32_O_3_ | 5,6-Epoxy-8,11,14-eicosatrienoic acid | HMDB0002190 | C14768 | 1.68 | 319[M-H]^-^,301[M-H-H_2_O]^-^,257[M-H-CH_2_O_2_]^-^,179[M-H-C_8_H_12_O_2_]^-^ | ↑** | ↑# | 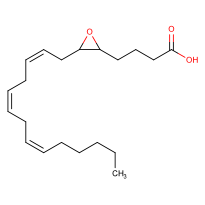 |
| 3.10_121.0661 | 122.073 | ESI- | M-H | C_8_H_10_O | 4-Ethylphenol | HMDB0029306 | C13637 | 1.38 | 121[M-H]^-^,103[M-H-H_3_O]^-^ | ↓** | ↓ | 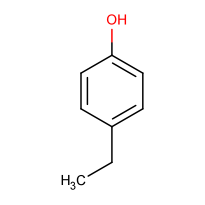 |

↑: The content of the model group was higher than that of the control group.

↓: The content of the model group was lower than that of the control group.

**↑:** The content of the treatment group was higher than that of the control group.

**↓:** The content of the treatment group was lower than that of the control group.

*: Compared with the control group, the content of markers in the model group changed significantly.

**: Compared with the control group, the content of markers in the model group changed [extremely](javascript:;) significantly.

**#**: Compared with the model group, the content of markers in treatment group changed significantly.

**##**: Compared with the model group, the content of markers in treatment group changed [extremely](javascript:;) significantly.


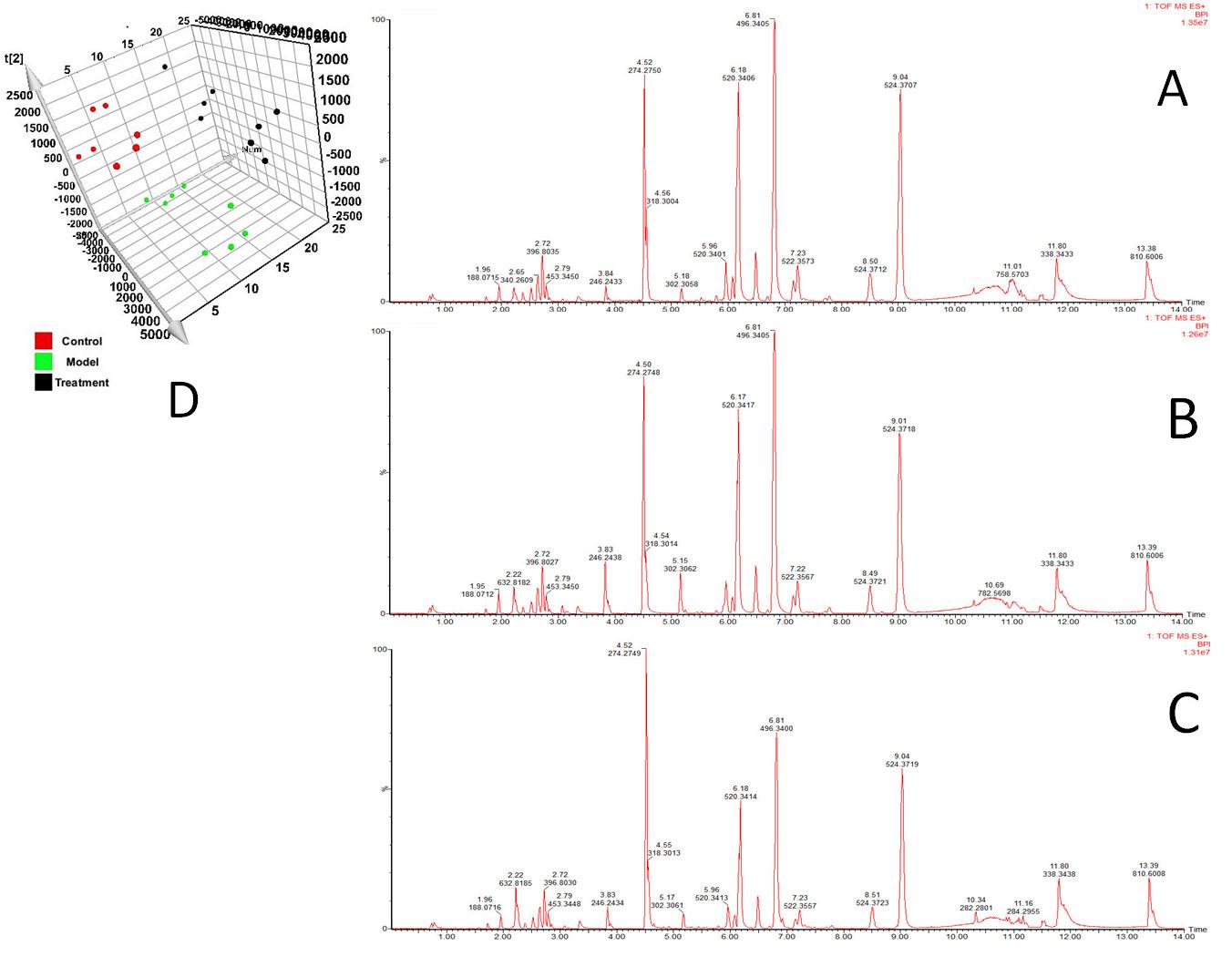


**Fig.S1.** 3D-Plot score plot of PCA analysis in serum of three groups of rats and UPLC-MS measured rat serum BPI ion map in positive ion mode. (A) Control group rat serum. (B) Model group rat serum. (C) Treatment group rat serum.(D) 3D-Plotof PCA


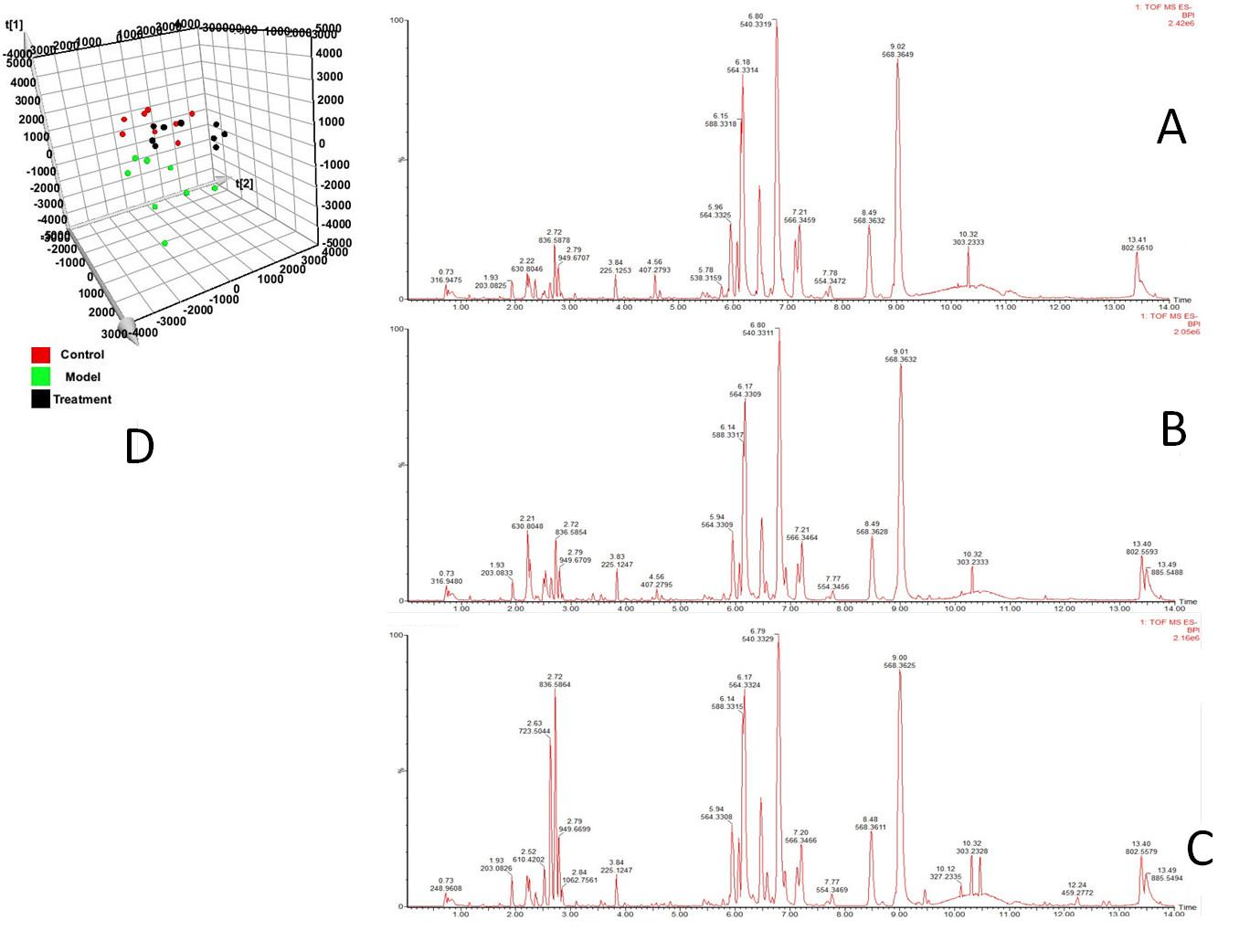


**Fig.S2.** 3D-Plot score plot of PCA analysis in serum of three groups of rats and UPLC-MS measured rat serum BPI ion map in negative ion mode. (A) Control group rat serum. (B) Model group rat serum. (C) Treatment group rat serum.(D) 3D-Plotof PCA.
